# Triterpene and Caffeoylquinic Acid Constituents Contribute to the Cognitive-Enhancing, but Not Anxiolytic, Effects of a Water Extract of *Centella asiatica* in Aged Mice

**DOI:** 10.3390/nu17193171

**Published:** 2025-10-08

**Authors:** Wyatt Hack, Lucas Kuhnau, Jesus Martinez, Luke C. Marney, Jaewoo Choi, Arshia R. Sohal, Seiji Koike, Thuan Nguyen, Claudia S. Maier, Amala Soumyanath, Nora E. Gray

**Affiliations:** 1 BENFRA Botanical Dietary Supplements Research Center, Oregon Health & Science University, Portland, OR 97239, USA; 2 Department of Neurology, Oregon Health & Science University, Portland, OR 97239-3098, USA; 3 Department of Chemistry, Oregon State University, Corvallis, OR 97331, USA; 4 Linus Pauling Institute, Oregon State University, Corvallis, OR 97331, USA; 5 School of Public Health, Oregon Health & Science University, Portland, OR 97201, USA

**Keywords:** triterpenes, caffeoylquinic acids, *Centella asiatica*, cognition, anxiety, aging

## Abstract

Background/objectives: A water extract of the plant *Centella asiatica* (CAW) has been shown to improve cognitive deficits in aged mice when administered for 5 weeks in drinking water. However, the contribution of the constituent compounds within CAW to the beneficial effects of the extract remains unelucidated. This study evaluated the effects of triterpene (TT) and caffeoylquinic acids (CQA) found within CAW, on learning, cognitive flexibility, memory, and anxiety-like behaviors in aged C57BL6 mice. Methods: Eighteen-month-old male and female C57BL6 mice were administered either TT, CQA, or the combination (TT+CQA) in their drinking water for a total of 5 weeks, at concentrations corresponding to their presence in CAW. During the final two weeks of treatment learning, executive function, memory, and anxiety were assessed. Results: Aged mice of both sexes showed significant improvement in learning when treated with TT and CQA separately and in combination. Treatment with TT also improved cognitive flexibility in aged mice of both sexes, but CQA and the combination of TT+CQA did not alter cognitive flexibility in aged male mice. There was no effect on recognition memory or anxiety in any of the treatment groups (TT, CQA, TT+CQA) relative to mice treated with the vehicle control although there was a trend towards improved recognition memory with TT treatment. Conclusions: These results suggest that the TT and CQA present in CAW likely contribute to its previously reported amelioration of age-related cognitive changes, especially in learning and cognitive flexibility, while other constituents may be responsible for CAW’s anxiolytic effects.

## 1. Introduction

An aging population and the prevalence of age-related health concerns are some of the most pressing issues today. The worldwide proportion of people over 65 nearly doubled between 1974 and 2024 [1]. By 2050, people in this age group are expected to reach 22% of the global population [2]. Along with the physical symptoms of aging, there is also concern about cognitive decline and quality of life in an increasingly aged population. Cognitive deficits in the aging population are well-studied, especially in the realms of executive function, memory, and spatial reasoning [3,4]. Mood changes are also well-documented, with anxiety symptoms occurring in as high as 56% of older adults [5]. These changes in cognition and mood can significantly affect the quality of life of elderly individuals, underscoring the need for effective and safe interventions for the prevention or treatment of these symptoms, and yet therapeutic options remain limited. This has contributed to a surge of interest among the elderly in complementary and alternative therapies, including botanically derived interventions [6]. The use of medicinal plants has shown many promising benefits in the realm of cognition and other health concerns relevant to an aging population [7,8], and these interventions often have fewer side effects than conventional drugs currently used for age-related cognitive or mood-related symptoms [9,10].

*Centella asiatica* (L.) Urban (CA) is an edible plant that has been used as a traditional medicine for more than 2000 years and has long been known for its therapeutic effects on cognitive function and memory [11]. CA preparations have been shown in clinical studies to reduce symptoms of anxiety and cognitive impairment [12,13]. CA is reported to have other neurobiological effects, such as the reduction in inflammation and oxidative stress [14,15], which are known to be important in the development of age-related disorders. Studies of CA in rodent models have also been promising, demonstrating improved performance in memory and learning tasks following oral administration [16,17,18]. This includes evidence that treatment with CA improves cognitive function in healthy adult male rats [19] and can mitigate the neurobehavioral effects of cerebral ischemic injury in male rats [20]. Our own group has repeatedly shown that oral treatment with a water extract of CA (CAW, 2–10 g/L) can significantly improve cognitive deficits in both healthy aged mice as well as mouse models of Alzheimer’s disease [21,22,23,24,25,26,27]. Most recently, we have shown that 10 g/L CAW likewise improves measures of cognition, including learning, recognition memory, and cognitive flexibility in aged C57BL6 mice of both sexes and attenuates anxiety-related behavior in aged female mice [28].

The chemical complexity of CAW has made producing a standardized preparation very challenging. Further adding to this challenge is an unclear understanding of the contribution of its constituent compounds to the beneficial effects evoked by CAW. The triterpene (TT) and caffeoylquinic acid (CQA) compounds in CAW both appear to participate in mediating the effects of the extract in both healthy and beta-amyloid exposed primary neurons as well as in mouse models of Alzheimer’s disease [29,30,31]. Additionally, one of these same triterpene compounds have been shown to improve motor dysfunction and offered neuroprotective effects in a rat model of parkinsonism [32]. Similarly, treatment with one of the CQA from CAW protected against cognitive decline in a mouse model of Alzheimer’s disease [33]. While this evidence supports the hypothesis that TT and CQA are the major active compounds within CAW, they have not previously been studied in combination at the concentrations at which they are present in CAW. The study described here builds on previous work to address this important unanswered question of whether the administration of TT and CQA from CAW, as well as their combination (TT+CQA), can elicit the same improvements in learning, memory, cognitive flexibility, and anxiety-related behaviors as was seen in response to CAW in healthy aged C57BL6 mice. We hypothesized that TT and CQA may improve these outcomes separately but the combination of TT+CQA would elicit the most robust response.

## 2. Materials and Methods

### 2.1. Animals

Eighteen-month-old male and female C57BL6 mice were obtained from the National Institute of Aging Aged Rodent Colony and housed in a climate-controlled environment with a 12 h light/12 h dark cycle. Water and diet (AIN-93M; Dyets Inc., Bethlehem, PA, USA) were supplied ad libitum, except during the odor discrimination reversal learning testing when food was restricted at night and resupplied in the afternoon following testing. All methods were performed in correspondence with the National Institutes of Health (NIH) guidelines for the Care and Use of Laboratory Animals and were approved by the Institutional Animal Care and Use Committee of the Portland VA Medical Center (ACORP protocol #4469-23). Mice were randomly assigned to treatment groups with 19–20 animals per group (9–10 of each sex in each treatment condition).

### 2.2. Compound Treatments

TT, CQA, and TT+CQA treatments were prepared at their relative concentrations in CAW (10 g/L), based on analysis using liquid chromatography with multiple reaction monitoring mass spectrometry (LC-MRM-MS) [34] of a batch of CAW (BEN-CAW-8) previously shown to improve cognition and reduce anxiety in aged mice [28]. Concentrations of 4 TT and 8 CQA compounds in CAW are given in Supplementary Table S1.

The TT and CQA compounds measured in BEN-CAW-8 were purchased from Wuhan ChemFaces Biochemical Co., Ltd. (Wuhan, Hubei, China): asiaticoside [CAS 16830-15-2], madecassoside [CAS 34540-22-2], asiatic acid [CAS 464-92-6], madecassic acid [CAS 18449-41-7], 5-caffeoylquinic acid [CAS 906-333-2], 4-caffeoylquinic acid [CAS 905-99-7], 3-caffeoylquinic acid [CAS 327-97-9], 1,3-dicaffeoylquinic acid [CAS 30964-13-7], 1,5-dicaffeoylquinic acid [CAS 19870-46-3], 3,4-dicaffeoylquinic acid [CAS 14534-61-3], 3,5-dicaffeoylquinic acid [CAS 2450-53-5], and 4,5-dicaffeoylquinic acid [CAS 32451-88-0]. Their identity and purity were verified by ^1^H Nuclear Magnetic Resonance (NMR) analysis at the Botanical Core of the BENFRA Botanical Dietary Supplements Research Center. Most compounds had no detectable impurity peaks in their NMR spectra, suggesting close to 100% purity. However, 5-caffeoylquinic acid and 3,4-dicaffeoylquinic acid were calculated to be 90% and 93% pure, respectively, based on impurity peaks in the NMR spectra. The purity of these compounds was taken into account in preparing the target concentrations of the compounds to match their content in CAW.

Initially, stock mixtures containing 1000× of the final required concentration of 4 TT or 8 CQA were prepared by weighing and mixing the required weights in 50% aqueous ethanol. Mixtures containing 500× the final concentrations of TT, CQA, or TT+CQA were then made by mixing the 1000× TT or CQA solutions with an equal volume of 50% ethanol, or by mixing equal volumes of the 1000× TT and 1000× CQA mixtures. The 500× mixture of CQA in 50% ethanol was a clear solution whereas the 500× TT and TT+CQA mixtures were suspensions. The 500× mixtures were vortexed to ensure homogeneity and aliquoted into 2 mL portions. These, along with 2 mL aliquots of 50% ethanol, were stored at −80 °C until use. Drinking water solutions for administration to mice were prepared by diluting the relevant 2 mL aliquots of 500× TT, CQA, and TT+CQA mixtures or 50% ethanol (vehicle control) to 1 L with deionized water. The final drinking water preparations were all clear solutions containing TT, CQA, TT+CQA, or no compounds (vehicle control), in 0.1% ethanol. The TT solution administered was prepared to contain madecassoside (344.6 mg/L), asiaticoside (145.8 mg/L), madecassic acid (14.0 mg/L), and asiatic acid (7.9 mg/L). The CQA solution was prepared to contain 3-caffeoylquinic acid (72.1 mg/L), 5-caffeoylquinic acid (34.0 mg/L), 4-caffeoylquinic acid (30.3 mg/L), 3,4-dicaffeoylquinic acid (25.1 mg/L), 3,5-dicaffeoylquinic acid (20.1 mg/L)), 4,5-dicaffeoylquinic acid (21.5 mg/L), 1,3-dicaffeoylquinic acid (24.9 mg/L), and 1,5-dicaffeoylquinic acid (38.6 mg/L). The triterpene and caffeoylquinic acid (TT+CQA) solution was prepared to contain the TT and CQA at concentrations listed for the separate TT and CQA solutions. The vehicle control consisted of water containing ethanol (0.1% *v*/*v*).

Compound solutions or the vehicle control were administered to mice as drinking water, and were replaced twice weekly, i.e., every 3 or 4 days. The compound concentrations listed above represent ‘theoretical values’ based on the method of preparation of the solutions. For each treatment condition, the concentration and stability of those compounds in freshly prepared drinking water solutions, and when stored at 4 °C, was measured in two independently prepared samples and monitored over 7 weeks (Supplementary Table S2). Additionally, the stability of the compounds in the drinking water bottles placed in the animal cages was also assessed in a representative sample after 4 days of exposure, which was the longest interval of time between replacing the solutions (Supplementary Table S3).

**Figure 1:**
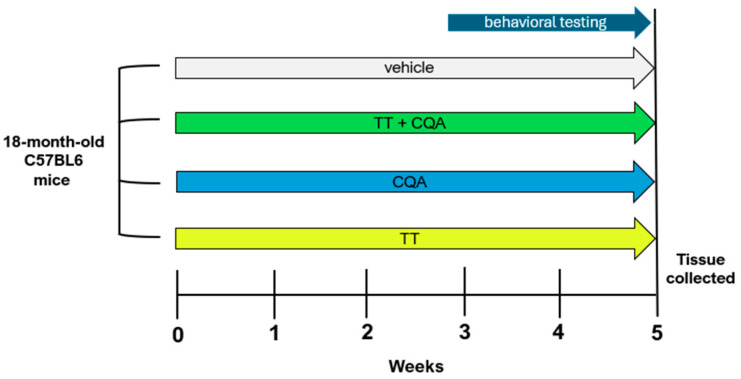
Experiment timeline. Eighteen-month-old male and female C57BL/6 mice were treated for 5 weeks total with continuous access to either TT, CQA, TT+CQA, or vehicle control (*n* = 20; 9–10 of each sex per treatment condition) in the drinking water. Behavioral testing started after 2 weeks of access and continued for 2 weeks, with mice euthanized in the 5th week.

The treatment paradigm is outlined in Figure 1. Eighteen-month-old male and female mice were treated with TT, CQA, and TT+CQA in their drinking water or with the vehicle control for a total of 5 weeks (*n* = 19–20 per condition; 9–10 of each sex). Mice were housed 2–4 animals per cage and water consumption was monitored for each cage.

### 2.3. Behavioral Testing

In the final 2 weeks of treatment, mice underwent behavioral testing including odor discrimination reversal learning (ODRL) testing, novel object recognition testing (NORT), and open field (OF) testing.

ODRL: ODRL evaluates learning and cognitive flexibility [35] and was performed as previously reported [22]. The test occurs following training to dig for a food reward and is divided into two stages. In the acquisition stage, mice were presented with two cups, one that contains dried beans and one with string. In every trial, one digging material was scented with a mint odor and the other with vanilla. Pairings were randomly alternated and the location of the baited cup (right vs. left) was balanced over the experiment. The number of trials for each mouse to reach criteria (8 correct trials in a bout of 10) was recorded. Fewer trials required to reach the criteria in the acquisition phase is indicative of enhanced learning. After a mouse reached the criteria in the acquisition phase, the shift phase was immediately initiated. In the shift phase, mice were presented with two cups containing the same digging materials and odors but, in this phase, the dried beans were always baited regardless of odor so the mice had learn to associate the food reward with the digging material and ignore the odor. Again, criteria was defined as 8 correct trials in any bout of 10 and trials to criteria was recorded. Fewer trials required to reach criteria in the shift phase is indicative of enhanced cognitive flexibility. Mice were food restricted the night before each phase of the ODRL to motivate the animals.

NORT: NORT tests recognition memory [36]. It was performed as previously described [27]. The test occurs in 2 phases (training and testing) that begin after mice complete two 10 min habituation sessions in the arena. During testing, animals were exposed to two identical objects for 10 min, once an hour over a 3 h window. Then, the testing sessions occurred 2 h and 24 h after the final training session. During testing, one of the identical objects was replaced with a novel object and the time spent exploring the familiar and novel objects over 5 min was evaluated via a camera placed above the arena, interfaced with an ANYmaze video tracking system (Stoelting Co, Wood Dale, IL, USA). A discrimination index was calculated using the equation:

$$\frac{\left(\mathrm{time}\;\mathrm{exploring}\;\mathrm{novel}\;\mathrm{object}-\mathrm{time}\;\mathrm{exploring}\;\mathrm{familiar}\;\mathrm{object}\right)}{\left(\mathrm{time}\;\mathrm{exploring}\;\mathrm{novel}\;\mathrm{object}+\mathrm{time}\;\mathrm{exploring}\;\mathrm{familiar}\;\mathrm{object}\right)}\times 100$$

At 24 h, a second novel object (distinct from the object used in the 2 h test) was used along with the familiar one from training. Again, the time spent exploring the familiar and novel objects was evaluated. Decreased time with the novel object reflects impairments in recognition memory. If the animal did not interact with either object for at least 3 s it was determined to be non-participating and not included in the analysis for that test. A table of non-participating animals in each treatment group is found in Supplementary Table S4. For this reason, we obtained data from fewer than 9–10 of each sex per treatment group in the NORT.

OF: The OF test is an assessment of anxiety as well as overall activity [37]. In the OF test, each mouse was placed into a square arena (38 cm × 38 cm × 64 cm high, constructed of white acrylonitrile butadiene styrene) for a 10 min open field session. A camera mounted above the arena, interfaced with a video tracking system (Any-maze, Erie, PA, USA), captured time in the center (s) as well as time immobile (s). Increased time in the center and reduced time immobile were associated with decreased measures of anxiety.

### 2.4. Statistical Analyses

To determine if there were differences across treatment groups and the vehicle control, a series of linear models were fit using the main effects of treatment group, sex, and their interaction terms. A likelihood ratio test was performed to determine if there were any moderating effects of sex on treatment. If there was no strong evidence of such effects, the simpler additive model was used to ascertain group differences. Right-skewed outcomes were log-transformed. If the non-constant variance assumption did not hold, then Huber–White standard errors were implemented. Comparisons between treatments and the vehicle control were performed within sex, and *p*-values were adjusted using a multivariate t correction. Analyses were performed in R (version 4.4.1) using the emmeans (version 1.11.2) package for group comparisons.

## 3. Results

### 3.1. Treatment with TT and CQA Separately and in Combination Improves Learning in Aged Mice

**Figure 2:**
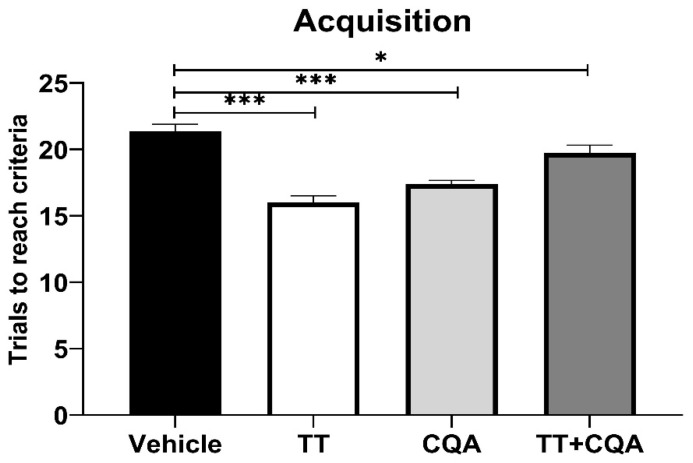
Treatment with TT and CQA separately and in combination improves learning in aged mice. During the acquisition phase of the ODRL test, aged mice treated with either TT, CQA, or TT+CQA exhibited a significant reduction in the number of trials needed to reach criteria when compared to vehicle-treated animals. *n* = 19–20 per condition. Columns indicate the average for that treatment group with error bars reflecting standard error of the mean. *** *p* ≤ 0.001, * *p* ≤ 0.05.

The ODRL test assesses learning and executive function, specifically cognitive flexibility. The acquisition phase evaluates learning, with more trials indicating poorer learning. There was no significant interaction effect between sex and treatment in the acquisition phase, so data from males and females are shown together. Aged mice treated with TT, trials 16.0 +/− 1.5 trials (mean +/− SEM), or with CQA, 17.4 +/− 1.1 trials, at their relative concentrations in 10 g/L CAW, showed significant reductions in the number of trials to reach criteria compared to vehicle-treated animals, 21.4 +/− 1.5 trials (*p* < 0.001; Figure 2). Treatment with TT+CQA, 19.8 +/− 1.6 trials, also significantly improved performance in the acquisition phase relative to vehicle-treated animals (*p* = 0.03).

### 3.2. Treatment with TT and CQA Separately Improved Cognitive Flexibility in Aged Mice of Both Sexes, but Not When Administered in Combination in Aged Male Mice

**Figure 3:**
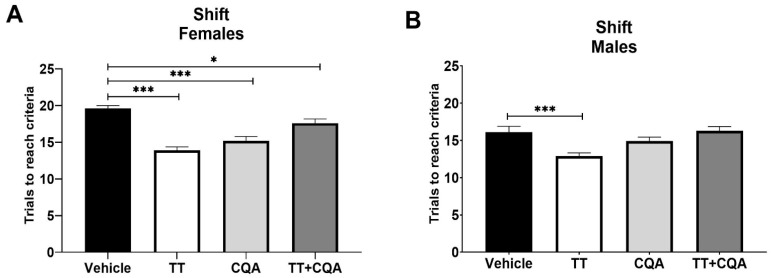
Treatment with TT and CQA separately improved cognitive flexibility in aged mice of both sexes, but not in combination when administered to aged male mice. (**A**) Aged female mice treated with either TT, CQA, or TT+CQA showed a significant improvement in the shift phase of the ODRL test relative to aged females treated with the vehicle control. *n* = 10 per condition. (**B**) Male mice treated with TT also showed significance relative to vehicle-treated mice during the shift phase. *n* = 9–10 * *p* < 0.05, *** *p* ≤ 0.001. Columns indicate the average for that treatment group with error bars reflecting standard error of the mean.

The shift phase of ODRL assesses cognitive flexibility. There was a significant interaction between sex and treatment response in this phase (*p* = 0.03), so males and females are shown separately. We observed effects of similar significance in the females as in the acquisition phase data, with female mice in the TT, 13.9 +/− 0.5 trials, and CQA, 15.2 +/− 0.6 trials, treatment groups showing a significant improvement (*p* < 0.001) in performance during the shift phase relative to animals in the vehicle group, 19.6 +/− 0.4 trials, as did the female TT+CQA group, 17.6 +/− 0.6 trials (*p* = 0.03). Male mice treated with TT also showed significant improvement in performance, 12.9 +/− 0.4 trials, relative to vehicle-treated mice, 16.1 +/− 0.8 trials, during the shift phase (*p* < 0.001, Figure 3); however, male mice treated with either CQA alone, 14.9 +/− 0.5 trials, or the combination of TT+CQA, 16.3 +/− 0.6 trials, did not display significant improvements (Figure 3).

### 3.3. TT, CQA, and TT+CQA Do Not Significantly Improve Recognition Memory in Aged Mice

**Figure 4:**
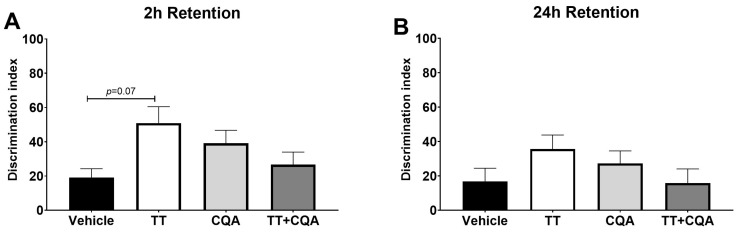
Treatment with TT and CQA separately and in combination do not significantly improve recognition memory in aged mice. There was no significant difference in the discrimination index for any of the treatment groups (TT, CQA, TT+CQA) relative to the vehicle control in either the (**A**) 2 h or the (**B**) 24 h retention tests. *n* = 11–17 per condition in the 2 h test and 15–18 per condition in the 24 h test. Columns indicate the average for that treatment group with error bars reflecting standard error of the mean.

The Novel Object Recognition Test (NORT) assesses recognition memory by comparing the time spent with a familiar object versus a novel one, with less time spent exploring the novel object indicating a deficit in memory due to the exploratory nature of rodents. There was no evidence of an interaction effect of sex on treatment response in either the 2 h or 24 h test so data from both sexes are presented together. During the 2 h test (Figure 4A), there was no significant change in the discrimination index with any of the treatments (TT—50.9 +/− 9.6, CQA—39.2 +/− 7.5, and TT+CQA—26.7 +/− 7.3) groups relative to the vehicle control—19.0 +/− 5.3. However, there was evidence of a trend toward improved recognition memory in the TT-treated animals (*p* = 0.07). In the 24 h test there was no evidence of significant improvement in any of the treatment groups (TT—35.5 +/− 8.1, CQA—34.2 +/− 7.3, or TT+CQA—17.8 +/− 8.3 compared to the vehicle group—18.7 +/− 7.6 (Figure 4B). These data can also be found repesented as percent time with the novel object in Supplementary Figure S1.

### 3.4. Treatment with TT, CQA, or TT+CQA Does Not Significantly Reduce Anxiety or Affect Overall Mobility in Aged Mice

**Figure 5:**
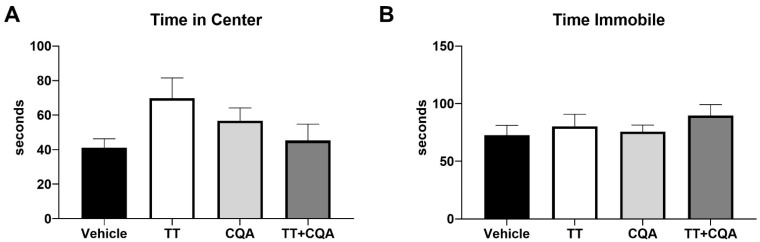
Treatment with TT, CQA, or TT+CQA does not significantly reduce anxiety or affect overall mobility. There was no effect of TT or CQA separately or in combination on (**A**) the time spent in the center or (**B**) the amount of time spent immobile relative to vehicle-treated mice in the OF test. *n* = 19–20 per condition. Columns indicate the average for that treatment group with error bars reflecting standard error of the mean.

The Open Field (OF) test is used to assess overall mobility and anxiety-related behaviors. Increased time in the center of the open field and reduced episodes of immobility suggest decreased anxiety. There was no evidence of an interaction effect between sex and treatment response in any of the OF endpoints, so data from males and females are shown together. Treatment with TT (70 +/− 17 s), CQA (57 +/− 10 s), or TT+CQA (45 +/− 13 s) did not significantly alter time spent in the center (Figure 5A) of the arena relative to vehicle-treated animals (41 +/− 8 s) and similarly did not significantly affect the amount of time spent immobile (TT: 80 +/− 15 s; CQA: 76 +/− 8 s; TT+CQA: 90 +/− 13 s; Figure 5B) relative to vehicle-treated mice (73 +/− 12 s).

## 4. Discussion

The prevalence of cognitive impairment and anxiety among the elderly makes clear the urgent need for therapies that can promote resilience to these age-related changes. Our group has previously reported that a water extract of the plant *Centella asiatica* (CAW) can improve learning, memory, and cognitive flexibility and decrease anxiety-related behavior in aged mice [28] when administered in their drinking water at 10 g/L. The present study investigated whether groups of constituent compounds from CAW, specifically TT and CQA, or the combination of the two, administered at concentrations equivalent to their abundance in 10 g/L CAW could elicit the same beneficial effects.

We found that TT, CQA, and TT+CQA all significantly improved learning relative to vehicle-treated mice as measured in the acquisition phase of ODRL and there was no interaction between sex and response to the treatments. This is in line with what was observed in response to CAW in our previous study [28] where there was also a significant improvement with CAW and no interaction between sex and response to the treatment.

Cognitive flexibility was measured in the shift phase of ODRL where an interaction between sex and response to treatment was observed. TT treatment improved cognitive flexibility in aged mice of both sexes, which is again consistent with the effect observed in response to CAW [28]. Interestingly the CQA and TT+CQA treatments only improved cognitive flexibility in female aged mice but did not elicit a significant response in male mice. This is distinct from our work with the entire extract where there was no difference in response based on sex [28]. Future studies are needed to test these conditions side by side in order to confirm these effects and clarify what other compounds within CAW might be important for this effect.

While our previous study showed that CAW treatment resulted in improved recognition memory [28], in the present study, none of the treatments with constituent compounds significantly improved performance in the NORT. This suggests that other compounds within the extract are necessary in order to elicit the full effect on recognition memory. It is interesting as well that in the previous study there was no interaction between sex and response to CAW in the NORT test but there was an interaction here in response to the individual compounds. There was a trend towards improved performance in the TT treated group during the 2 h test (*p* = 0.07) but in the 24 h test, this trend was no longer apparent (*p* = 0.45). The previous study with CAW demonstrated a significant improvement at 2 h and a trend towards improvement at 24 h [28]. This suggests that TT may be important for the effects of CAW on short term memory but cannot explain the full effect of the extract. It is also interesting to note that the effect of CAW in the previous study was likewise more robust at 2 h as compared to 24 h which may indicate that the extract is less effective for long-term memory enhancement.

There was no improvement in anxiety-related behavior in response to any of the compound treatments in this study. This too is distinct from what was reported previously in response to CAW [28], where CAW resulted in decreased time immobile for both sexes and reduced in the center only for female mice. These findings suggest that TT and CQA may not participate in the anxiolytic effect of CAW although further investigation is needed to more fully understand the differential response to CAW between males and females.

Comparing the data from this study to the earlier CAW study suggests that the magnitude of the response to CAW might be greater than the responses to TT or CQA and is quite likely greater than the response to TT+CQA (Supplementary Figure S2A). In the previously published study [28], mice treated with 10 g/L CAW required, on average, only 60% of trials compared to controls to reach criteria in the acquisition phase of the ODRL test, whereas in the present study mice treated with TT required 75%, CQA 81%, and TT+CQA 92% of the number of trials needed by vehicle controls. Additionally, in that previously published study [28], female mice treated with 10 g/L CAW required, on average, 64% of the trials needed by controls to reach criteria in the shift phase of the ODRL test, whereas here the number of trials needed with compound treatments was 98% of control with TT+CQA, 77% with TT, and 85% with CQA. For male mice, the response observed with CAW in the previous study was also 64% of the trials needed by controls, whereas in the present study, the number of trials compared to control was 72% with TT, 83% with CQA, and 91% with TT+CQA (Supplementary Figure S2B,C). Taken together, the ODRL data indicates that TT in particular are likely to play an important role in the effects of CAW on learning and memory, although CQA may also participate, with neither group producing as large a response as CAW. A larger future study with CAW and compound treatments side-by-side would allow for direct statistical comparison of the treatment conditions as well as enable a more in-depth analysis of potential sex differences. It would also be of interest to compare plasma levels of the TT and CQA following each of the treatments to evaluate the role of bioavailability in the observed differences in activity.

The findings from this study support previous reports in the literature about the cognitive-enhancing effects of TT and CQA compounds. TT are often considered to be “the active compounds” of CA [38,39] and CA-containing dietary supplements are often standardized to these compounds. There is substantial evidence for the cognitive-enhancing effects of TT in the literature [11]. Oral administration of the single TT, asiatic acid, in neonatal mice attenuated glutamate-induced deficits in spatial memory [40]. Our own group similarly demonstrated cognitive improvements following oral asiatic acid treatment, showing improved association memory in 5xFAD mice although interestingly this effect was only observed in female animals [28]. Asiaticoside has likewise been reported to improve cognitive performance ameliorating deficits in spatial memory in a mouse model of diabetes-induced cognitive impairment [41] and a rat model of vascular dementia [42]. Madecassoside has also been shown to improve spatial memory in both a D-galactose mouse model of cognitive impairment [43] as well as in a rat model of traumatic brain injury [44].

CQA have only more recently been linked to the neurological benefits of CA [29,30,31,45]. We have previously reported that five weeks of treatment with the same combination of CQA evaluated here improved associative memory in 6-month-old animals from the 5xFAD mouse model of β-amyloid accumulation when administered in the diet at concentrations proportional to their abundance in CAW [30]. Similarly, a CQA-rich extract of purple sweet potato likewise improved memory in the SAMP8 mouse model of accelerated senescence [46]. CQA administered individually can also improve cognition. Four months of treatment with chlorogenic acid, (3-CQA), administered in the diet, beginning at 7 weeks of age, also improved recognition and spatial memory in 5xFAD mice [33]. Those same domains of cognitive function were also improved in the APP/PS2 mouse model of β-amyloid accumulation following 21 weeks of dietary treatment with 5-CQA, also known as neochlorogenic acid, beginning at 10 weeks of age [47].

Chlorogenic acid has also been reported to improve spatial memory in a mouse model of LPS-induced cognitive impairment [48] and was able to prevent spatial memory impairments following sleep deprivation in mice [49]. There is even evidence of chlorogenic acids improving cognitive function in human trials of both healthy individuals as well as those with mild cognitive impairments [50,51].

It is interesting that in the present study, TT, CQA, and TT+CQA compound treatments did not elicit the same magnitude of response as CAW. To establish the true concentrations of TT and CQA administered to the animals, drinking water solutions were analyzed using LC-MRM-MS, using a previously described method [34]. Analysis of freshly prepared TT, CQA, and TT+CQA solutions in drinking water conducted after one week of storage at 4C showed that, particularly for the TT, their measured concentrations were lower than the intended ones matching CAW 10 g/L (Supplementary Table S2). This may have been due to poor solubility in the 500× stock mixtures, resulting in some losses during the preparation and dilution process. Decomposition of stock solutions during storage may also have occurred. However, for the TT glycosides (MS and AS), if hydrolysis had occurred there would be a corresponding increase in the levels of the aglycones (MA and AA), which was not the case. For 4,5-diCQA and 5-CQA the two CQA which had lower than expected concentrations at week 1, it is possible that some decomposition had already occurred during storage of the stock solutions, as described below for the diluted samples. This lower than intended concentration may account for the weaker activity of TT and TT+CQA mixtures compared to CAW 10 g/L. However, CAW has shown robust cognitive effects at lower doses [21,22,23,24,25,26,27] containing lower amounts of TT. Therefore, a lack of activity of TT and CQA in given biological tests or sexes either signifies that they are not the active compounds of CAW for those conditions, or that they require the presence of other CAW components in order to exert their activity.

Due to the high cost of the pure compounds, and in order to conserve resources, we also stored some of the prepared drinking water solutions for up to 7 weeks at 4 °C before use in mouse cages, although the majority of animals received solutions stored for no more than 4 weeks from preparation. Stability analysis over 7 weeks indicated that while the TT were relatively stable, the CQA, in particular the diCQA, decreased in concentration on storage (Supplementary Table S2). This was likely due to hydrolysis of the caffeoyl esters as previously reported [52,53]. Over a four-day period in an animal cage at ambient temperature (Supplementary Table S3), the majority of CQA remained at 80% or more of the starting concentration in the drinking water. Again, the TT showed better stability than the CQA, with some TT concentrations even increasing slightly over 4 days, likely due to evaporation of the water. Although some level decomposition occurred in CQA over 4 days in the animal cages, fresh drinking water containing the compounds was provided every 3–4 days, with CQA at the higher concentration.

The lack of effect of TT and CQA on anxiety in the present study is surprising given reports in the literature of their anxiolytic effects. CQA have been shown to reduce anxiety in both fly and mouse model systems. Our group has shown the same CQA evaluated in the present study, also at their relative concentrations in CAW, can promote resilience to chronic stress-induced behavioral changes in *Drosophila melanogaster* [45]. In mice, chlorogenic acid has been shown to prevent anxiety-like behaviors induced by chronic stress in mice [54]. There is an even more substantial literature associating TT with anxiolytic effects. A study evaluating a standardized extract of triterpenoids from *Centella asiatica* reported a decrease in anxiety-related behaviors in both chronically stressed and unstressed mice [55]. There is also evidence of the anxiolytic effects of TT when given individually. Asiatic acid has been shown to improve performance in the elevated plus maze in healthy Sprague Dawley rats [56] as well as in a mouse model of aluminum chloride toxicity [57]. Asiaticoside has likewise been shown to elicit anti-anxiety effects in both healthy mice [58] and those subjected to chronic unpredictable stress [59]. Madecassoside has similarly been reported to have anxiolytic properties reducing anxiety-related behaviors in a mouse model of L-isoaspartyl methyltransferase deficiency-induced neurodegeneration [60]. These discrepancies between the previous reports and the present study may be due to varying doses used in the studies or it may reflect a differential response to administering several TT and CQA compounds together compared to individual compounds. Future work is needed to clarify this finding.

It is interesting that in the present study, the combination of TT+CQA did not evoke a stronger effect than the TT and CQA treatments administered separately in any of the tests. In fact, in the acquisition phase of ODRL, TT+CQA resulted in less significant a change from the vehicle-treated mice than either TT or CQA (*p* = 0.03 for TT+CQA as compared to *p* < 0.001 for TT and CQA) and the same was true for the female mice in the shift phase (also *p* = 0.03 for TT+CQA as compared to *p* < 0.001 for TT and CQA). This suggests the possibility that the combination of TT+CQA inhibits the effects of the compound groups individually. Moreover, when taken together with the results of the previous study with showing a very robust effect of the entire CAW extract [28], the results seen here in response to TT+CQA indicate the likely presence of additional compounds within the CAW that either relieve the inhibitory effects of the TT and CQA or potentiate their effects so that the improved behavioral effect is seen. A similar blunted effect of TT+CQA as compared to TT alone was seen in the number of differentially expressed genes identified in a transcriptomics analysis of mouse primary cortical neurons treated with the same TT, CQA, and TT+CQA conditions [61]. We have also found that in *Drosophila melanogaster*, TT+CQA was unable to prevent stress-induced behavioral changes whereas CQA alone did [45]. Future studies are needed to more fully understand the interactions between TT and CQA compounds as well as to identify other compounds within CAW that might alter this interaction and contribute to the beneficial effects of CAW.

## 5. Conclusions

Overall, this study demonstrates that TT and CQA contribute to the effects of CAW on learning and cognitive flexibility but do not appear to be the main drivers of the effects on anxiety-related behavior. Despite the relatively small sample size and lack of direct comparison to CAW, this is the first study looking into the effects of both TT and CQA and their combination, and we plan to rectify these existing limitations with future studies. Along with evaluating the compounds investigated here alongside CAW, further work sub-fractionating the extract or generating extracts with individual compounds removed could help clarify the compounds most important for the effects of *Centella asiatica* on cognition and anxiety in aged mice.

## Data Availability

The data in this study are available upon request from the corresponding author.

## Supplementary Materials

The following supporting information can be downloaded at: https://www.mdpi.com/article/10.3390/nu17193171/s1.

## Abbreviations

The following abbreviations are used in this manuscript:

CA	*Centella asiatica*
TT	TT
CQA	Caffeoylquinic Acid
ODRL	Odor Discrimination Reversal Learning
OF	Open Field
NORT	Novel Object Recognition Test
