# Supplementary material for: Triterpene and Caffeoylquinic Acid Constituents Contribute to the Cognitive-Enhancing, but Not Anxiolytic, Effects of a Water Extract of Centella asiatica in Aged Mice
Source: Nutrients. 2025 Oct 8;17(19):3171. doi: 10.3390/nu17193171 (PMC12526379; doi:10.3390/nu17193171)
Supplement: Supplementary file 1 [file nutrients-17-03171-s001.zip › nutrients-3874554-supplementary.pdf]

SUPPLEMENTARY MATERIALS

Supplementary Table S1 Quantified content of 12 bioactive compounds present in *Centella asiatica* aqueous extract , BEN-CAW-8, from Yang et al, 2023

Compound	Amount in BEN-CAW-8 (mg/g)
5-CQA	3.40 ± 0.19
4-CQA	3.03 ± 0.21
3-CQA	7.21 ± 0.42
1,3-DiCQA	2.49 ± 0.14
3,4-DiCQA	2.33 ± 0.16
3,5-DiCQA	2.01 ± 0.23
1,5-DiCQA	3.86 ± 0.30
4,5-DiCQA	2.15 ± 0.14
MS	34.46 ± 1.32
AS	14.58 ± 1.00
MA	1.40 ± 0.20
AA	0.79 ± 0.15

Supplementary Table S2: Stability of treatment solutions stored at 4C over 7 weeks – The stability of constituent compounds within each treatment solution stored at 4°C over 7 weeks was monitored by LC-MRM-MS in two independently prepared samples of the treatments. The theoretical equivalent of the concentration of a given compound within CAW (10 mg/mL) is also listed on the table.

	TT Sample 1 (ug/mL)					TT Sample 2 (ug/mL)			
	MS	AS	MA	AA		MS	AS	MA	AA
CAW Equivalent	344.6	145.8	14.0	7.9	CAW Equivalent	344.6	145.8	14.0	7.9
Week 1	199.7 ± 6.9	129.8 ± 5.3	7.1 ± 0.7	3.5 ± 0.3	Week 1	206.1 ± 3.6	140.5 ± 5.1	7.6 ± 0.6	3.6 ± 0.4
Week 3	206.5 ± 10.4	132.5 ± 6.8	7.9 ± 0.4	3.4 ± 0.3	Week 3	167.1 ± 31.6	112.9 ± 21	6.6 ± 1.4	2.9 ± 0.5
Week 5	210.6 ± 11.6	135.7 ± 8.2	8 ± 1.1	3.7 ± 0.5	Week 5	190.3 ± 19	125.6 ± 20.9	6.5 ± 0.8	1.7 ± 0.3
Week 7	196 ± 13.1	127.3 ± 10	7.2 ± 0.5	2.8 ± 0.4	Week 7	210.2 ± 14.1	135.9 ± 11.4	6.6 ± 0.7	1.2 ± 0.2

	CQA Sample 1 (ug/mL)							
	5-CQA	4-CQA	3-CQA	1,3 DiCQA	1,5 DiCQA	3,5 DiCQA	3,4 DiCQA	4,5 DiCQA
CAW Equivalent.	34.0	30.3	72.1	24.9	38.6	20.1	23.3	21.5
Week 1	21.8 ± 2.4	29.2 ± 3	66.5 ± 7	22.4 ± 1.9	38.1 ± 3	18 ± 1.7	21.7 ± 1.7	16.7 ± 1.2
Week 3	19.6 ± 1.7	26.6 ± 2.1	59.6 ± 5	19.9 ± 1.2	32.9 ± 1.8	15.4 ± 1.1	17.3 ± 1	11.1 ± 0.5
Week 5	19.7 ± 1.5	26.1 ± 1.9	54.4 ± 4	17.6 ± 0.9	26.1 ± 1.2	11.7 ± 0.6	11.5 ± 0.5	3.8 ± 0.2
Week 7	18.7 ± 1.5	23.2 ± 1.8	43.3 ± 3.4	15.1 ± 1	18.4 ± 1.3	7.2 ± 0.5	8.6 ± 0.6	2.2 ± 0.1

	CQA Sample 2 (ug/mL)							
	5-CQA	4-CQA	3-CQA	1,3 DiCQA	1,5 DiCQA	3,5 DiCQA	3,4 DiCQA	4,5 DiCQA
CAW Equivalent	34.0	30.3	72.1	24.9	38.6	20.1	23.3	21.5
Week 1	23.8 ± 1.8	32.3 ± 2.2	72.8 ± 5.2	24 ± 1.9	40.2 ± 3.6	19.1 ± 1.8	22.2 ± 1.7	16.8 ± 1.4
Week 3	21.8 ± 1.9	30.2 ± 2.1	68.1 ± 5.2	22.2 ± 1.7	36.8 ± 2.9	17.4 ± 1.3	19.4 ± 1.4	12.2 ± 0.8
Week 5	22.2 ± 2.5	28.8 ± 2.8	59.6 ± 5.9	20 ± 1.8	29.7 ± 2.8	13.8 ± 1.3	15.9 ± 1.4	6.9 ± 0.6
Week 7	22.6 ± 1.6	28.6 ± 2	59.4 ± 4.3	19.9 ± 1.6	29.4 ± 2.4	13.6 ± 1.1	15.6 ± 1.2	6.4 ± 0.5

	TT+CQA Sample 1 (ug/mL)											
	5-CQA	4-CQA	3-CQA	1,3 DiCQA	1,5 DiCQA	3,5 DiCQA	3,4 DiCQA	4,5 DiCQA	MS	AS	MA	AA
CAW Equivalent	34.0	30.3	72.1	24.9	38.6	20.1	23.3	21.5	344.6	145.8	14.0	7.9
Week 1	20.3 ± 2	26.6 ± 2.5	60.9 ± 5.7	20.5 ± 1.3	35 ± 2.2	16.4 ± 1.3	20 ± 1.2	15.6 ± 0.8	212.4 ± 9.7	140.8 ± 6.4	9.6 ± 4.1	3.9 ± 0.8
Week 3	16.6 ± 4.6	24.3 ± 3.7	51.7 ± 11.2	14.1 ± 8.2	29.4 ± 4.1	12.5 ± 3.6	15 ± 3.9	9.2 ± 3.5	210.8 ± 13.5	139.3 ± 8.2	6.8 ± 0.5	3.3 ± 0.3
Week 5	20.1 ± 2.1	25.3 ± 2.7	53.4 ± 5.6	17.9 ± 1.5	26.9 ± 2.2	12.4 ± 1.2	13.4 ± 1.2	5.6 ± 0.5	218.7 ± 14.5	145.3 ± 9.6	7.2 ± 0.6	2.7 ± 0.3
Week 7	17.4 ± 2	21.3 ± 2.1	38.9 ± 4.2	14.1 ± 1.1	16 ± 1.4	6.6 ± 0.6	8.4 ± 0.9	2.4 ± 0.3	197.5 ± 8.8	131 ± 5.8	6.3 ± 0.3	1.9 ± 0.2

	TT+CQA Sample 2 (ug/mL)											
	5-CQA	4-CQA	3-CQA	1,3 DiCQA	1,5 DiCQA	3,5 DiCQA	3,4 DiCQA	4,5 DiCQA	MS	AS	MA	AA
CAW Equivalent	34.0	30.3	72.1	24.9	38.6	20.1	23.3	21.5	344.6	145.8	14.0	7.9
Week 1	19.5 ± 1.6	26.2 ± 2	59.5 ± 4.7	20 ± 1.6	33.1 ± 3	15.2 ± 1.3	18.7 ± 1.6	14 ± 1.2	236.6 ± 13.3	156.1 ± 8.5	7.3 ± 0.7	3.8 ± 0.4
Week 3	18.3 ± 2.5	24.3 ± 3.1	54.5 ± 6.8	17.5 ± 2.2	29.4 ± 3.6	13.5 ± 1.7	15.5 ± 2	9.9 ± 1.4	218.3 ± 16.1	143.2 ± 10.5	5.5 ± 0.4	2.4 ± 0.3
Week 5	20 ± 2	24.1 ± 2.1	49.4 ± 4.5	18.5 ± 1.9	27.5 ± 3	12.3 ± 1.3	15.2 ± 1.4	7.1 ± 0.6	234.3 ± 15.8	154.2 ± 10	5.4 ± 0.8	1.4 ± 0.3
Week 7	15.9 ± 5.5	18.9 ± 6.2	38.4 ± 12	15.1 ± 5.3	22.6 ± 7.4	10.1 ± 3	12.1 ± 3.8	5.7 ± 1.5	190.1 ± 67.5	126.2 ± 43.1	4.8 ± 0.7	1 ± 0.2

Supplementary Table S3: Stability of treatment solutions in the animal cage over 4 days. The stability of constituent compounds within each treatment solution was monitored over 4 days of exposure in the animal cage by LC-MRM-MS. The theoretical equivalent of the the concentration of a given compound with in the CAW extract is also listed on the table.

	In Cage Stability (µg/mL)											
	4-CQA	5-CQA	3-CQA	1,3-DiCQA	3,4-DiCQA	3,5-DiCQA	1,5-DiCQA	4,5-DiCQA	MS	AS	MA	AA
CAW Equivalent	34.0	30.3	72.1	24.9	38.6	20.1	23.3	21.5	344.6	145.8	14.0	7.9
CQA Day 0	17.2 ± 3.7	13.5 ± 3	37.3 ± 7.9	13.3 ± 2.9	13.3 ± 3	10.8 ± 2.4	24.3 ± 5.4	9.8 ± 2.3				
CQA Day 4	15.6 ± 2	11.8 ± 1.6	31.5 ± 4.1	12 ± 1.6	10.4 ± 1.3	8.6 ± 1.1	20.1 ± 2.5	6 ± 0.8				
TT Day 0									153.3 ± 21.2	80.8 ± 11.3	6.1 ± 0.8	3.4 ± 0.4
TT Day 4									161.3 ± 23.5	85.3 ± 12.9	6.5 ± 1.1	3.1 ± 0.6
TT+CQA Day 0	15.4 ± 1.9	12.1 ± 1.7	32.8 ± 4.3	11.6 ± 1.6	11.4 ± 1.6	9.3 ± 1.3	20.9 ± 2.8	7.8 ± 1.1	156.4 ± 21	84.6 ± 11.4	3.6 ± 1.1	2.1 ± 0.5
TT+CQA Day 4	17.9 ± 4.8	13.2 ± 3.3	31.9 ± 8.1	13.4 ± 3.5	10.9 ± 3	8.6 ± 2.3	23.1 ± 6.1	5.4 ± 1.4	163.6 ± 14.1	88.4 ± 7.4	3.9 ± 0.9	2.3 ± 0.6

Supplementary Table S4: NORT participation – The number of animals in each treatment condition that participated in the NORT testing is listed. Non-participation was defined as an animal that did not explore either object for at least 3 seconds during the test period.

	Vehicle	TT	CQA	TT+CQA
2 hour test	7/10 females<br>7/9 males	7/10 females<br>4/10 males	9/10 females<br>8/10 males	10/10 females<br>5/10 males
24 hour test	7/10 females<br>7/9 males	10/10 females<br>8/10 males	8/10 females<br>8/10 males	10/10 females<br>8/10 males

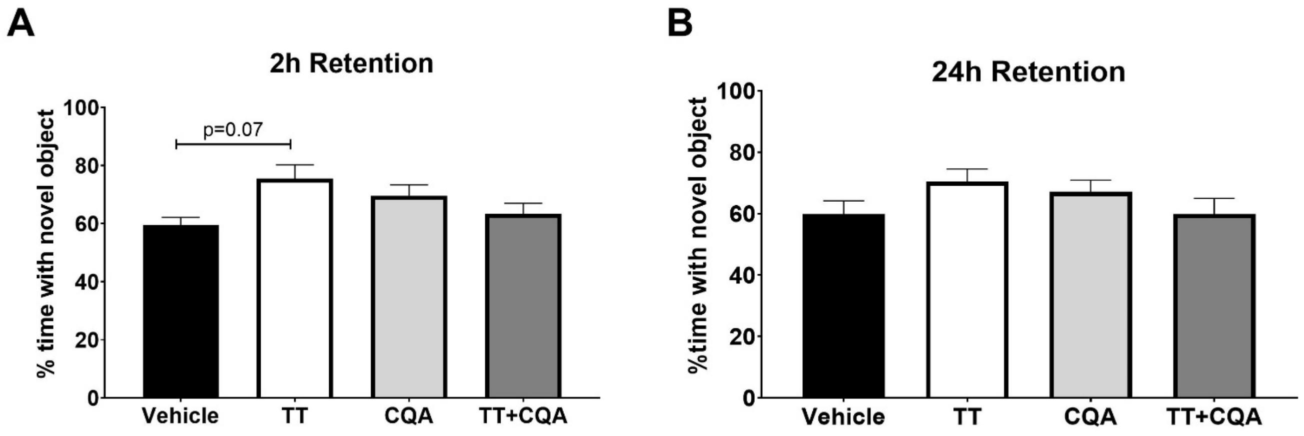

Supplementary Figure S1: Treatment with TTs and CQAs separately and in combination do not significantly improve recognition memory in aged mice. There was no significant difference in the time spent with the novel object in any of the treatment groups (TT, CQA, TT+CQA) relative to vehicle in either the A) 2-hour or the B) 24-hour retention tests. n=11-17 per condition in the 2 hour test and 15-18 per condition in the 24 hour test. Columns indicate average for that treatment group with error bars reflecting standard error of the mean.

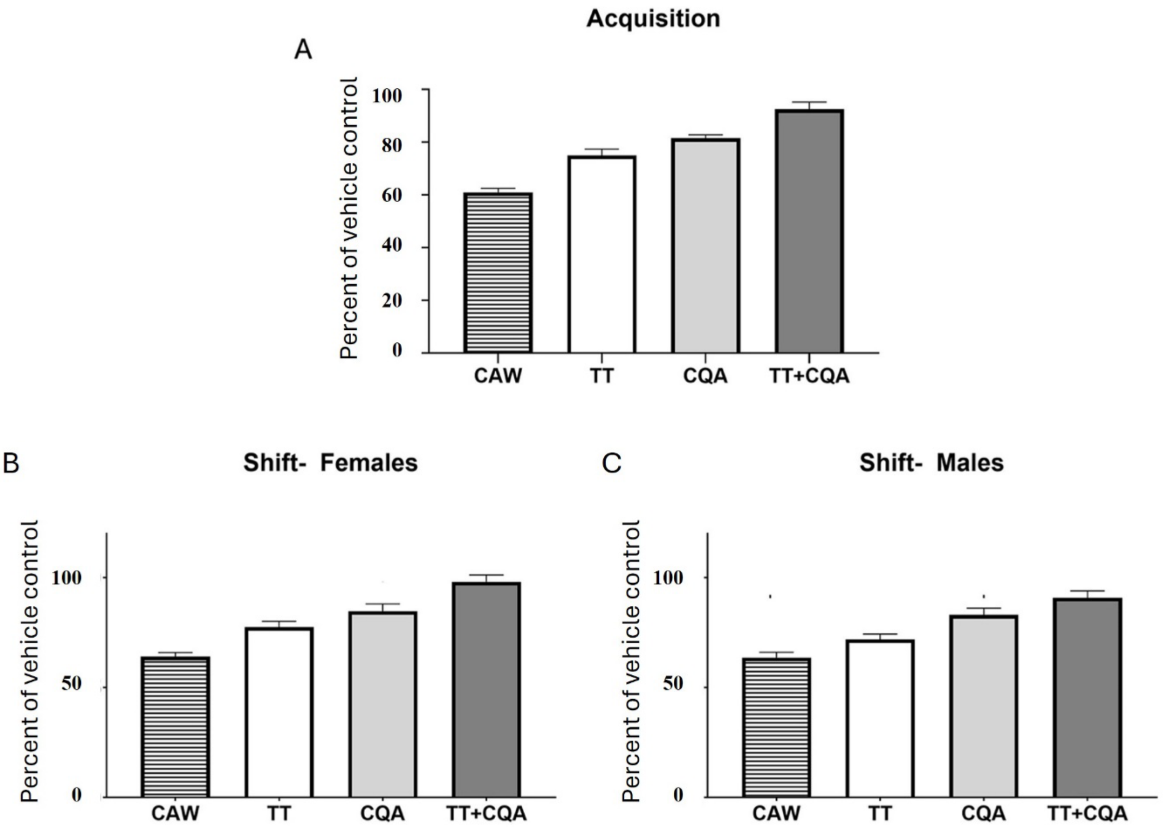

Supplementary Figure S2: Comparison of CAW response to compound response in the ODRL test. A) Data from mice of both sexes in the Acquisition phase of the ODRL from the present study where mice were treated with compounds at their relative concentration in 10 g/L CAW as well as the previously published study with 10g/L CAW (Gray et al. 2024) is plotted together as a percentage of the vehicle control response in each study. Data from the Shift phase of the ODRL is presented from both studies in the same way for both B) female and C) male mice. A lower percentage of vehicle indicates a stronger response to treatment.

References

Gray,N.E.; Hack, W.; Brandes, M.S.; Zweig, J.A.; Yang, L.; Marney, L.; Choi, J.; Magana, A.A.; Cerruti, N.; McFerrin, J., et al. Amelioration of age-related cognitive decline and anxiety in mice by Centella asiatica extract varies by sex, dose and mode of administration. *Front Aging* **2024**, *5*, 1357922, doi:10.3389/fragi.2024.1357922.

Yang, L.; Marney, L.; Magana, A.A.; Choi, J.; Wright, K.; McFerrin, J.; Gray, N.E.; Soumyanath, A.; Stevens, J.F.; Maier, C.S. Quantification of Caffeoylquinic Acids and Triterpenes as Targeted Bioactive Compounds of Centella asiatica in Extracts and Formulations by Liquid Chromatography Mass Spectrometry. *J Chromatogr Open* **2023**, *4*, doi:10.1016/j.jcoa.2023.100091.
